# Slow Metabolism–Driven Amplification of Hepatic PPARγ Agonism Mediates Benzbromarone‐Induced Obesity‐Specific Liver Injury

**DOI:** 10.1002/advs.202409126

**Published:** 2024-11-29

**Authors:** Guanting Li, Yourong Hu, Han Zhao, Ziyu Peng, Xin Shang, Jia Zhang, Kunxin Xie, Meiwei Li, Xiaohang Zhou, Qinyao Zhou, Kai Li, Fang Zhou, Heyao Wang, Zhijian Xu, Jiali Liu, Peng Sun

**Affiliations:** ^1^ The Affiliated Wuxi People's Hospital of Nanjing Medical University Wuxi People's Hospital Wuxi Medical Center Key Laboratory of Human Functional Genomics of Jiangsu Province Department of Biochemistry and Molecular Biology Nanjing Medical University Nanjing 211166 China; ^2^ State Key Laboratory of Drug Research Drug Discovery and Design Center Shanghai Institute of Materia Medica Chinese Academy of Sciences Shanghai 201203 China; ^3^ Key Laboratory of Drug Metabolism and Pharmacokinetics State Key Laboratory of Natural Medicines China Pharmaceutical University Nanjing 210009 China

**Keywords:** benzbromarone, drug‐induced liver injury, drug metabolism, obesity, PPARγ

## Abstract

Obesity and nonalcoholic fatty liver disease (NAFLD) are established risk factors for drug‐induced liver injury (DILI). The previous study demonstrates that benzbromarone (BBR), a commonly prescribed pharmaceutical agent for managing gout and hyperuricemia, exacerbates hepatic steatosis and liver injury specifically in obese individuals. However, the precise mechanism underpinning this adverse effect remains incompletely elucidated. Given the significance of BBR and its analogs in anti‐gout/hyperuricemia drug discovery, elucidating the mechanism by which BBR exacerbates obesity‐specific DILI warrants further investigation. In this study, through a combined multi‐omics, pharmacological, and pharmacokinetic approaches, it is found that BBR‐induced obesity‐specific DILI is primarily through the potentiation of peroxisome proliferator‐activated receptor gamma (PPARγ) signaling pathways. Further in vivo and in vitro pharmacokinetic analyses reveal that obese db/db mice exhibited a diminished capacity to metabolize BBR in their livers. This reduction leads to prolonged retention of BBR, subsequently resulting in chronic and sustained hepatic PPARγ agonism. This study demonstrates that a slow metabolism‐driven amplification of hepatic PPARγ agonism mediates BBR‐induced obesity‐specific hepatic steatosis and subsequent DILI, which also emphasizes the importance of the reduced hepatic drug metabolism capacity in patients with obesity or pre‐existing NAFLD in both clinical practice and drug discovery processes.

## Graphical abstract

Diminished hepatic drug metabolic capacity in obese individuals leads to the prolonged retention of BBR within the liver. This extended presence subsequently potentiates its agonistic effect on PPARγ, thereby exacerbating hepatic lipid accumulation and ultimately triggering an obesity‐specific drug‐induced liver injury (DILI).

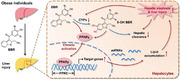

## 1 Introduction

Drug‐induced liver injury (DILI) is a prevalent contributor to hepatic damage; however, it has also led to the withdrawal of numerous medications from clinical use and has created a formidable challenge to the progress of novel drug discoveries. Distinct physicochemical properties of drugs, polymorphisms in genes coding for xenobiotic‐metabolizing enzymes (XMEs), and pre‐existing liver diseases are known to enhance the risk of DILI.^[^ ^1^ ^]^ Recently, nonalcoholic fatty liver disease (NAFLD) has increasingly been recognized as a predisposing factor for DILI, particularly in conjunction with certain medications.^[^ ^2^ ^]^ However, the precise mechanism that contributes to this adverse effect has yet to be fully elucidated.

Benzbromarone (BBR), an inhibitor of urate transporter 1 (URAT1), is one drug that has been linked to DILI. It has been used as a urate‐lowering therapy for gout and hyperuricemia since 1970;^[^ ^3^ ^]^ however, concerns regarding its potential hepatotoxicity have led to its withdrawal from the markets in Europe and the United States.^[^ ^4^ ^]^ Nevertheless, amid the growing prevalence of gout and hyperuricemia, particularly in East Asian countries like China and Japan, BBR continues to be a commonly prescribed urate‐lowering agent owing to the scarcity of alternative drug options. Consequently, the exploration of novel URAT1 inhibitors derived from the BBR scaffold remains a crucial research field in the discovery of anti‐gout/hyperuricemia drugs.^[^ ^5^ ^]^ Hence, elucidating the underlying mechanisms of BBR‐induced hepatotoxicity is imperative.

Previous studies have suggested that the hepatotoxic mechanism of BBR, like that of many other hepatotoxic drugs, is linked to its metabolism by hepatic drug‐metabolizing enzymes and the damage inflicted upon mitochondria.^[^ ^6^ ^]^ Nevertheless, initial studies incorporating preclinical drug safety assessment data have demonstrated that BBR exhibited negligible hepatotoxicity in the livers of healthy rats for up to 102 weeks of treatment.^[^ ^7^ ^]^ Thus, the traditional understanding of hepatic enzyme‐mediated drug metabolism may not fully elucidate the underlying mechanisms of BBR‐induced toxicity and liver injury.

We previously reported that BBR specifically exacerbates hepatic steatosis and liver injury in obese mice models, whereas no comparable liver damage is observed in healthy lean mice, suggesting a typical pattern of obesity‐specific DILI.^[^ ^8^ ^]^ Notably, BBR exhibited weak binding affinity for peroxisome proliferator‐activated receptor alpha (PPARα) and gamma (PPARγ), leading to enhanced expression of PPAR target genes.^[^ ^9^ ^]^ Hence, given the pivotal role of PPARs in lipid metabolism, we hypothesized that BBR may cause obesity‐related DILI by aggravating hepatic lipid accumulation.^[^ ^8^ ^]^ However, understanding the underlying mechanism still requires the resolution of two critical uncertainties: 1) Paradoxically, numerous studies have demonstrated that PPARα/γ agonists can mitigate the progression of NAFLD by enhancing hepatic fatty acid oxidation or peripheral insulin sensitivity.^[^ ^10^ ^]^ Therefore, whether the obesity‐specific DILI induced by BBR can be solely attributed to its dual agonistic activity targeting PPARα/γ remains uncertain; and 2) Compared to thiazolidinediones or fibrates, BBR is a relatively weak dual agonist for PPARα/γ; therefore, a remaining unanswered question is how BBR elicits severe hepatic steatosis in obese individuals.

To address these unresolved concerns, we used multi‐omics, pharmacological, and pharmacokinetic approaches to investigate the mechanistic effects of BBR on hepatic steatosis and liver injury in obese db/db mice and primary hepatocytes. Our study revealed that obese db/db mice showed diminished hepatic drug metabolic capacity, which resulted in prolonged retention of BBR within the liver. This extended presence subsequently potentiated its agonistic effect on PPARγ, thereby exacerbating hepatic lipid accumulation and ultimately triggering an obesity‐specific DILI.

## 2 Results

### 2.1 Benzbromarone Aggravates Lipid Accumulation in Primary Cultured Hepatocytes with Enhanced PPAR Signaling Pathways

**Figure 1:**
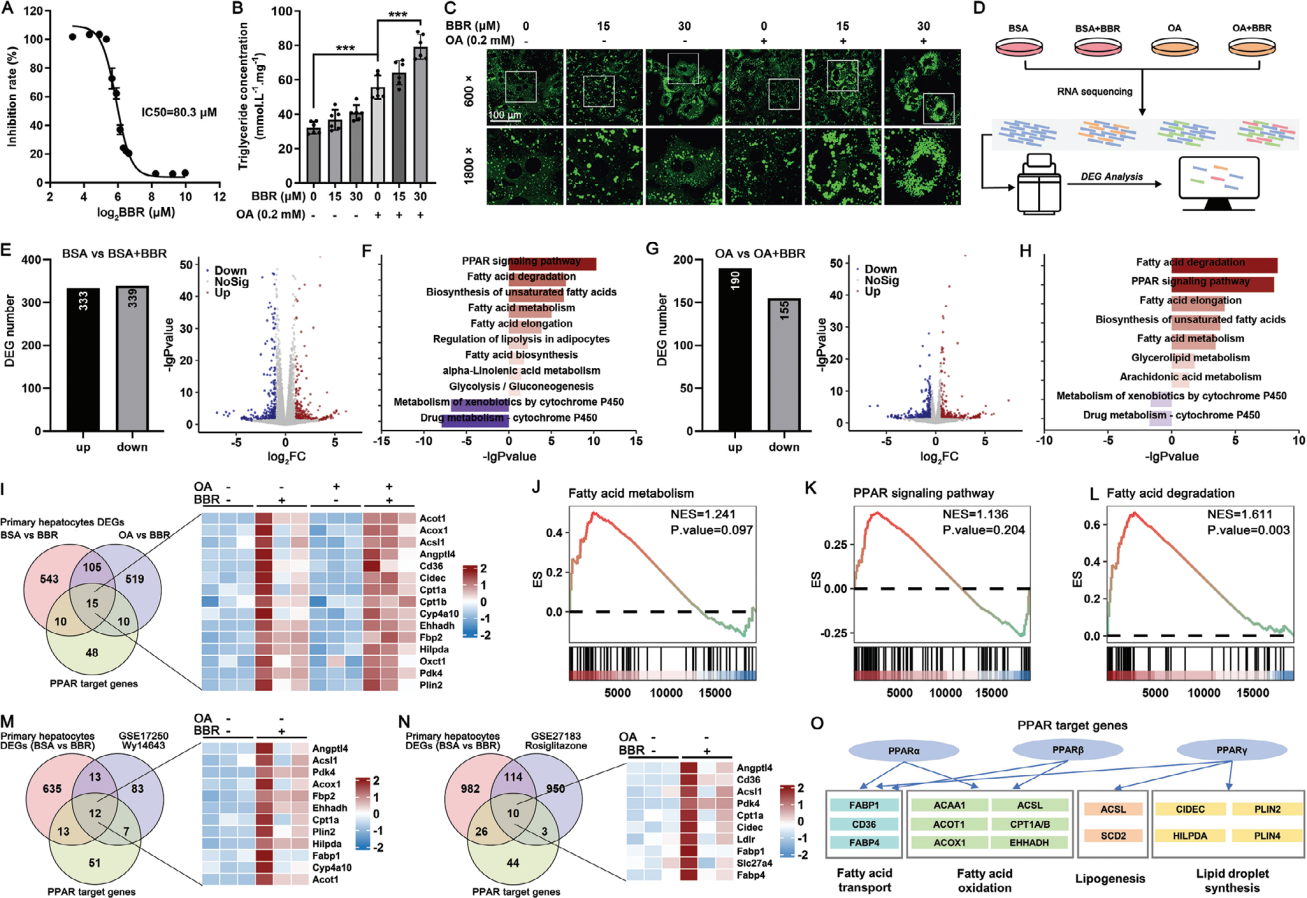
Benzbromarone (BBR) aggravates lipid accumulation in primary cultured hepatocytes by enhancing PPAR signaling pathways. A) Primary cultured mouse hepatocytes were incubated with different doses of BBR for 24 h, and the cell viability was measured by CCK8 assay to calculate the 50% inhibitory concentration (IC50). B) After primary hepatocytes were incubated with BBR/oleic acid (OA) as indicated for 16 h, intracellular triglyceride concentrations were measured, ***p < 0.001, *n* = 6. C) BODIPY staining in different groups; images are shown under 600× or 1800× magnification, scale bar = 100 µm. D) Flow scheme of RNA sequencing. E–H) Differentially expressed genes (DEGs), volcano plot, and KEGG enrichment in hepatocytes treated with BBR in the presence and absence of OA. I) Intersection and heatmap of DEGs in hepatocytes treated with BBR in the presence and absence of OA, against a predefined set of PPAR target genes. J–L) GSEA enrichment in BBR‐treated hepatocytes. M,N) Intersection and heatmap of DEGs in BBR‐treated hepatocytes compared to public transcriptomic datasets and PPAR target genes. O) BBR‐stimulated DEGs related to lipid metabolism in the PPAR signaling pathways.

We first tested the cytotoxicity of BBR in primary cultured hepatocytes and chose 15 and 30 µm as non‐toxic dosages in further experiments (**Figure** **1**A). Intracellular triglyceride concentration measurements (Figure 1B) and visualization of lipid droplets by BODIPY dye (Figure 1C) showed that BBR obviously enhanced lipid accumulation in oleic acid (OA)‐treated hepatocytes. We investigated the underlying mechanisms by conducting high‐throughput RNA sequencing analysis in hepatocytes exposed to 15 µm BBR for 16 h in either the presence or absence of 0.2 mm oleic acid (OA) (Figure 1D). We identified 333 upregulated and 339 downregulated DEGs in BBR‐treated hepatocytes compared to the control group (Figure 1E), and the upregulated DEGs indicated enrichment of multiple pathways related to lipid metabolism, whereas the downregulated DEGs were enriched in cytochrome P450 pathways (Figure 1F). Incubation of hepatocytes with BBR and OA revealed a pattern of signal transduction comparable to that seen in hepatocytes treated solely with BBR (Figure 1G,H).

A comparative analysis of DEGs in hepatocytes treated with BBR, both in the presence and absence of OA, against a predefined set of PPAR target genes (Table s3 , Supporting Information) revealed 15 genes in the intersection (Figure 1I). GSEA enrichment revealed that these 15 genes were enriched in fatty acid metabolism, PPAR signaling pathway, and fatty acid degradation (Figure 1J–L), further confirming the critical role of PPAR signaling pathways in BBR‐induced hepatic lipid accumulation. When compared with publicly available transcriptomic datasets utilizing the specific PPARα agonist Wy14643 and the PPARγ agonist rosiglitazone, our findings revealed that BBR elicited a comparable pattern of upregulated gene expression (Figure 1M,N). This observation suggests that BBR induces a dual agonistic effect on both PPARα and PPARγ, thereby regulating hepatic lipid metabolism (Figure 1O).

### 2.2 Benzbromarone Exacerbates Hepatic Steatosis in db/db Mice by Amplification of PPAR Signaling Pathways

**Figure 2:**
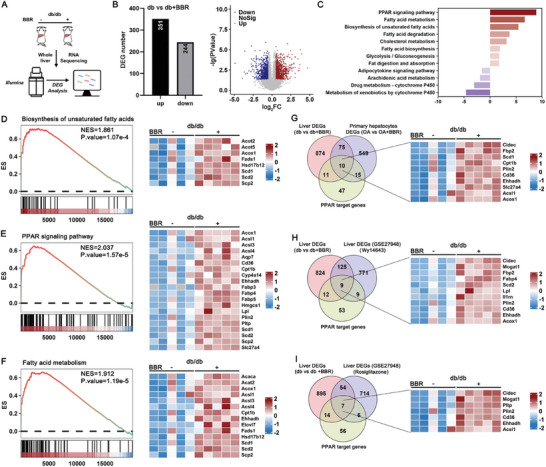
Benzbromarone exacerbates hepatic steatosis in db/db mice by amplification of PPAR signaling pathways. A) Flow scheme of RNA sequencing of livers from db/db mice. B) Differentially expressed genes (DEGs) and volcano plots for livers from BBR‐treated db/db mice compared to the vehicle control group. C) KEGG enrichment depicting upregulated DEGs. D–F) GSEA enrichment analysis and corresponding heatmaps depicting upregulated DEGs. G–I) Venn diagrams and heatmaps depicting upregulated DEGs in the livers of BBR‐treated db/db mice compared to BBR‐treated primary mouse hepatocytes cultured in vitro, livers from Wy14643‐treated mice, and livers from rosiglitazone‐treated mice.

We further validated the effects of BBR on PPAR activation observed in primary cultured hepatocytes by examining liver tissues excised from db/db mice that had been treated with BBR for 4 weeks and subjected to transcriptome sequencing (**Figure** **2**A). A total of 351 upregulated and 244 downregulated DEGs were identified and subsequently depicted in volcano plots (Figure 2B). KEGG and GSEA enrichment analyses suggested that BBR‐induced hepatic steatosis in db/db mice most possibly occurred via triggering of PPAR signaling pathways (Figure 2C–F). The findings aligned with the in vitro effects of BBR on OA‐treated primary hepatocytes (Figure 2G). Contrasting the effects of BBR with those observed in public datasets employing livers from mice administered either a specific PPARα agonist (Wy14643) or PPARγ agonist (rosiglitazone) confirmed that BBR elicited an upregulation of numerous shared PPAR target genes, further underscoring the dual agonistic potential of BBR toward both PPARα and PPARγ (Figure 2H,I).

### 2.3 Lipidomic Analysis Indicates Enhanced Lipid Synthesis in Benzbromarone‐Treated Hepatocytes

**Figure 3:**
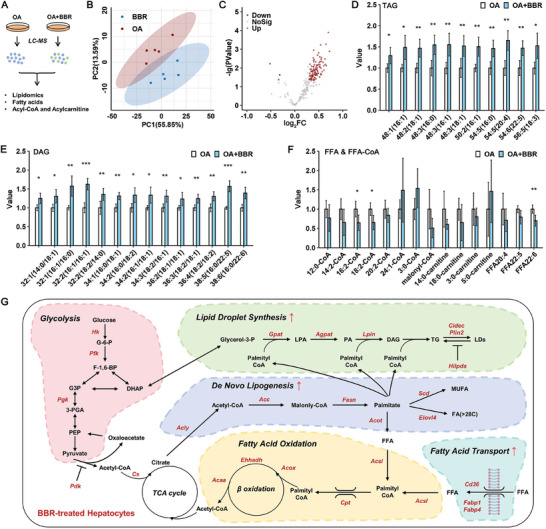
Lipidomic analysis indicates enhanced lipid synthesis in benzbromarone‐treated hepatocytes. A) Flow scheme of lipidomic analysis. B) Principal component analysis (PCA) between OA‐treated hepatocytes and those cotreated with OA and BBR. C) Volcano plot representing the levels of lipid species shows significant changes between the two groups. D) Levels of individual species of triglycerides (TAG), E) diacylglycerol (DAG), F) free fatty acids (FFA), and fatty acyl‐CoAs (FFA‐CoA), *p < 0.05, **p < 0.01, ***p < 0.001, *n* = 5. G) Network diagram illustrating the alterations in lipid metabolism pathways within BBR‐treated hepatocytes, with genes exhibiting upregulation highlighted in red font. TCA cycle, tricarboxylic acid cycle.

Unbiased lipidomic analysis (**Figure** **3**A) revealed that BBR co‐incubation clearly altered the lipid composition in OA‐treated primary hepatocytes (Figure 3B,C), as indicated by a pattern of increased levels of triacylglycerol (TAG, Figure 3D), as well as diacylglycerol (DAG, Figure 3E), whereas the levels of some free fatty acids (FFAs) and fatty acyl‐coenzyme A (FFA‐CoA) were reduced (Figure 3F).

The integration of RNA sequencing and lipidomic data incontrovertibly demonstrated an augmentation of lipid accumulation, encompassing de novo lipogenesis, TAG synthesis, and lipid droplet formation, in hepatocytes subjected to BBR treatment. This enhancement likely stems from the PPARγ agonistic activity of BBR. Although upregulation of select genes pivotal for fatty acid oxidation (such as Cpt, Acox, Ehhadh, and Acaa), potentially linked to PPARα activity, was also observed in BBR‐treated hepatocytes, the diminished levels of FFAs and FFA‐CoAs indicate that this upregulation may not be sufficient to mitigate BBR‐induced hepatic steatosis, as depicted in Figure 3G.

### 2.4 Inhibition of PPARγ Mitigates Benzbromarone‐Induced Hepatic Lipid Accumulation In Vitro

**Figure 4:**
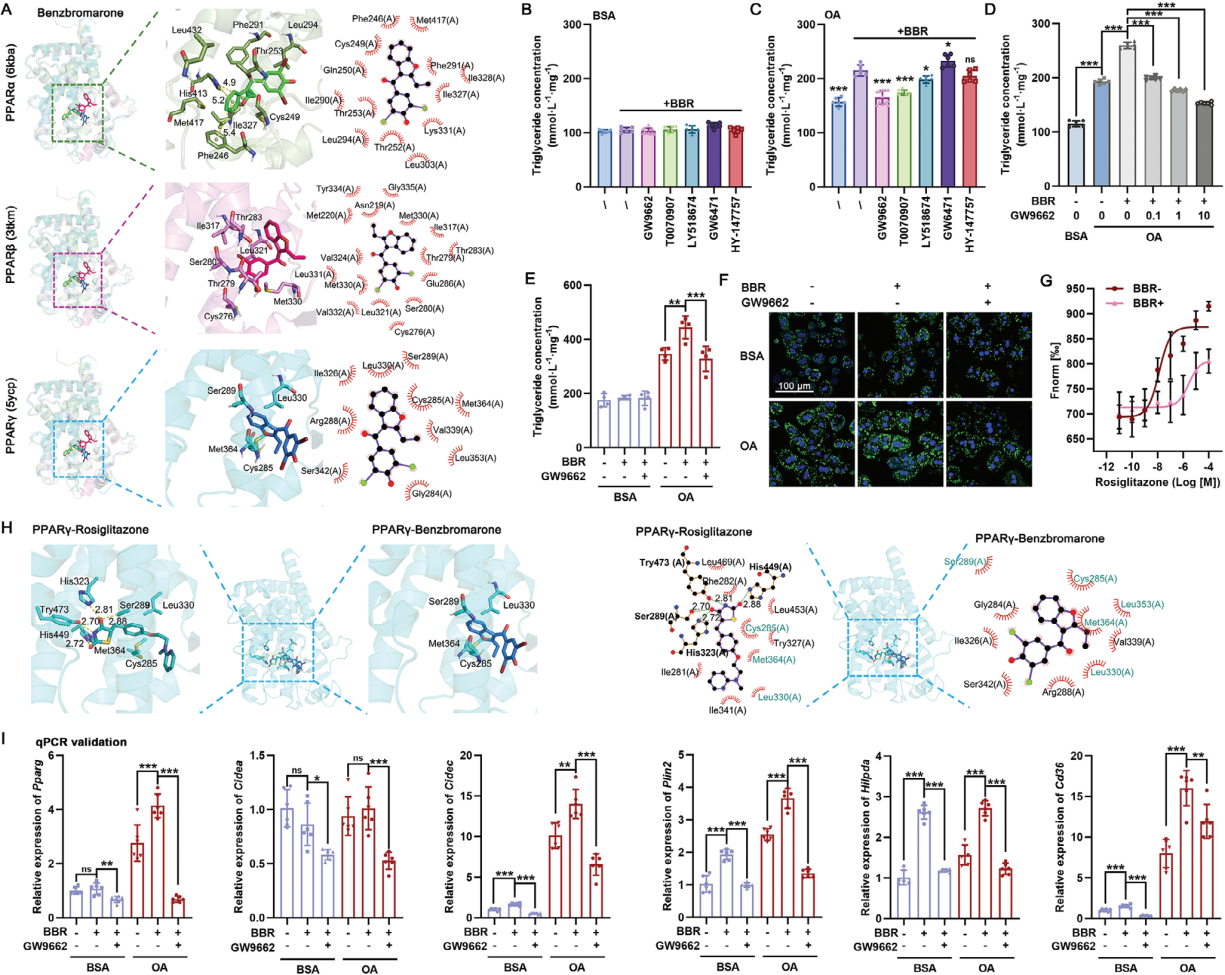
Inhibition of PPARγ mitigates benzbromarone‐induced hepatic lipid accumulation in vitro. A) Molecular docking of BBR with PPARα, PPARβ/δ, and PPARγ using Glide. B,C) Intracellular triglyceride levels in primary hepatocytes treated with BBR and different PPAR modulators. D–F) Intracellular triglyceride levels and BODIPY staining in hepatocytes treated with BBR and GW9662. G) MST experiment measuring the affinity between rosiglitazone and PPARγ in the presence/absence of BBR. H) Comparison of the PPARγ binding sites of BBR and rosiglitazone. The green residues are amino acids that show the same hydrophobic interactions with BBR and rosiglitazone. I) The mRNA expression of PPARγ target genes in primary hepatocytes after different treatments. *p < 0.05, **p < 0.01, ***p < 0.001, *n* = 6.

We clarified the precise selectivity of BBR toward different PPARs using Glide software to dock BBR to PPARα, PPARβ/δ, and PPARγ. The docking outcomes revealed that BBR achieved the most favorable docking score with PPARγ (−7.58 kcal·mol^−1^), suggesting a preferential binding affinity toward this receptor subtype (**Figure** **4**A; Table S4, Supporting Information). We also calculated the binding free energies of BBR to PPARα, PPARβ/δ, and PPARγ using the MM/GBSA method, and the results were consistent with the docking scores, thereby reinforcing the notion that BBR exhibits the strongest interaction with PPARγ (−25.55 kcal·mol^−1^) and the weakest with PPARα (−7.33 kcal·mol^−1^) and emphasizing the preferential binding of BBR to PPARγ rather than to PPARα. We validated the in‐silico findings by subjecting primary hepatocytes to co‐incubation experiments involving BBR in conjunction with various PPAR modulators, both in the presence and absence of OA. Specifically, the inclusion of GW9662 (a dual inhibitor of PPARα/γ), T0070907 (a PPARγ‐specific inhibitor), and LY518674 (a PPARα agonist) led to a notable reduction in triglyceride levels within BBR‐treated hepatocytes in the presence of OA. Conversely, the application of GW6471 (a PPARα inhibitor) and HY‐147757 (a PPARβ/δ inhibitor) failed to mitigate the BBR‐mediated increase in triglyceride levels (Figure 4B,C). The administration of different doses of GW9662, coupled with BODIPY staining, provided additional pharmacological evidence that reinforced the specific interaction between BBR and PPARγ (Figure 4D–F).

The results of MST assays demonstrated the competitive nature of BBR against the specific PPARγ agonist, rosiglitazone (Figure 4G). Our comparison of the docked conformations of rosiglitazone and BBR showed that the TZD head group of rosiglitazone forms a classical hydrogen bonding network with the AF‐2 helical region of PPARγ (Ser289, His323, His449, and Tyr473),^[^ ^11^ ^]^ but no hydrogen bonding is formed between BBR and the PPARγ. Both BBR and rosiglitazone undergo hydrophobic interactions with Met364, Leu330, Cys285, Leu353, and Ser289, showing that some of their binding sites overlap when binding PPARγ (Figure 4H). Although rosiglitazone formed an additional hydrogen bonding network with PPARγ to form a more stable binding than BBR (−9.64 kcal·mol^−1^ vs −7.58 kcal·mol^−1^), we hypothesize that BBR and rosiglitazone may have a similar competitive PPARγ binding site. Furthermore, GW9662 co‐incubation significantly diminished the BBR‐induced upregulation of typical PPARγ target genes (Figure 4I). These observations underscore the specificity of the interaction of BBR with PPARγ, thereby elucidating its role in promoting hepatic lipid accumulation.

### 2.5 Hepatic Knockdown of PPARγ Alleviates Benzbromarone‐Induced Liver Injury in db/db Mice

**Figure 5:**
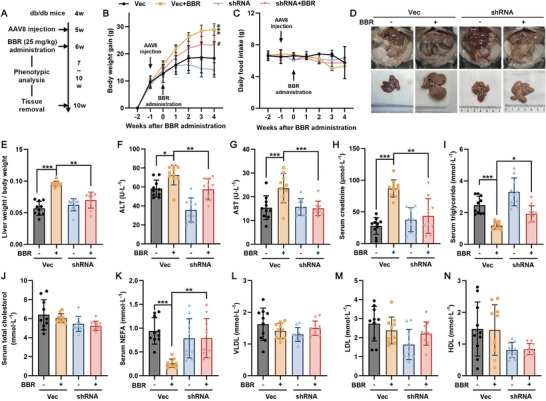
Hepatic knockdown of PPARγ alleviates benzbromarone‐induced liver injury in db/db mice. A) Flow scheme of BBR administration in db/db mice. B) Body weight gain, ***p < 0.001 compared to the vehicle‐treated AAV8‐Vec group; ^#^ *p* < 0.05 compared to the BBR‐treated AAV8‐Vec group, *n* = 10. C) Daily food intake (g·day^−1^ per mouse). D) Representative images of the liver and other abdominal organs. E) Liver weight normalized by body weight in each group. The serum levels of F) alanine transaminase (ALT), G) aspartate transaminase (AST), H) creatinine, I) triglyceride, J) total cholesterol (TC), K) non‐esterified fatty acid (NEFA), L) very low‐density lipoprotein (VLDL), M) low‐density lipoprotein (LDL), and N) high‐density lipoprotein (HDL). *p < 0.05, **p < 0.01, ***p < 0.001 as indicated, *n* = 10.

After 4 weeks of BBR administration (**Figure** **5**A), BBR‐induced increases in liver weight, serum transaminases, and creatinine levels were obviously alleviated in db/db mice by PPARγ knockdown using AAV8‐PPARγ‐shRNA versus with AAV8‐Vec, whereas the reductions in serum triglyceride and non‐esterified fatty acid (NEFA) levels by BBR treatment were reversed, indicating improved liver function (Figure 5B–K). In addition, daily food intake (Figure 5C); kidney, spleen, and abdominal adipose weights (Figure S1A–C, Supporting Information); serum TC (Figure 5J), VLDL, LDL, and HDL (Figure 5L–N) levels; and triglyceride and TC levels in adipose tissue (Figure S1D,E, Supporting Information) were less altered. Notably, the 4‐week BBR treatment of db/db mice significantly decreased their fasting plasma glucose levels and improved their glucose tolerance (Figure S1F–J, Supporting Information). Nonetheless, pyruvate tolerance and insulin sensitivity were concurrently diminished (Figure 1F–N, Supporting Information), indicating that this reduction in blood glucose levels is likely attributable to compromised hepatic glucose production caused by liver damage. This finding aligns with our previous research.^[^ ^8^ ^]^

### 2.6 Knockdown of PPARγ Restores Benzbromarone‐Induced Aggravation of Hepatic Steatosis in db/db Mice

**Figure 6:**
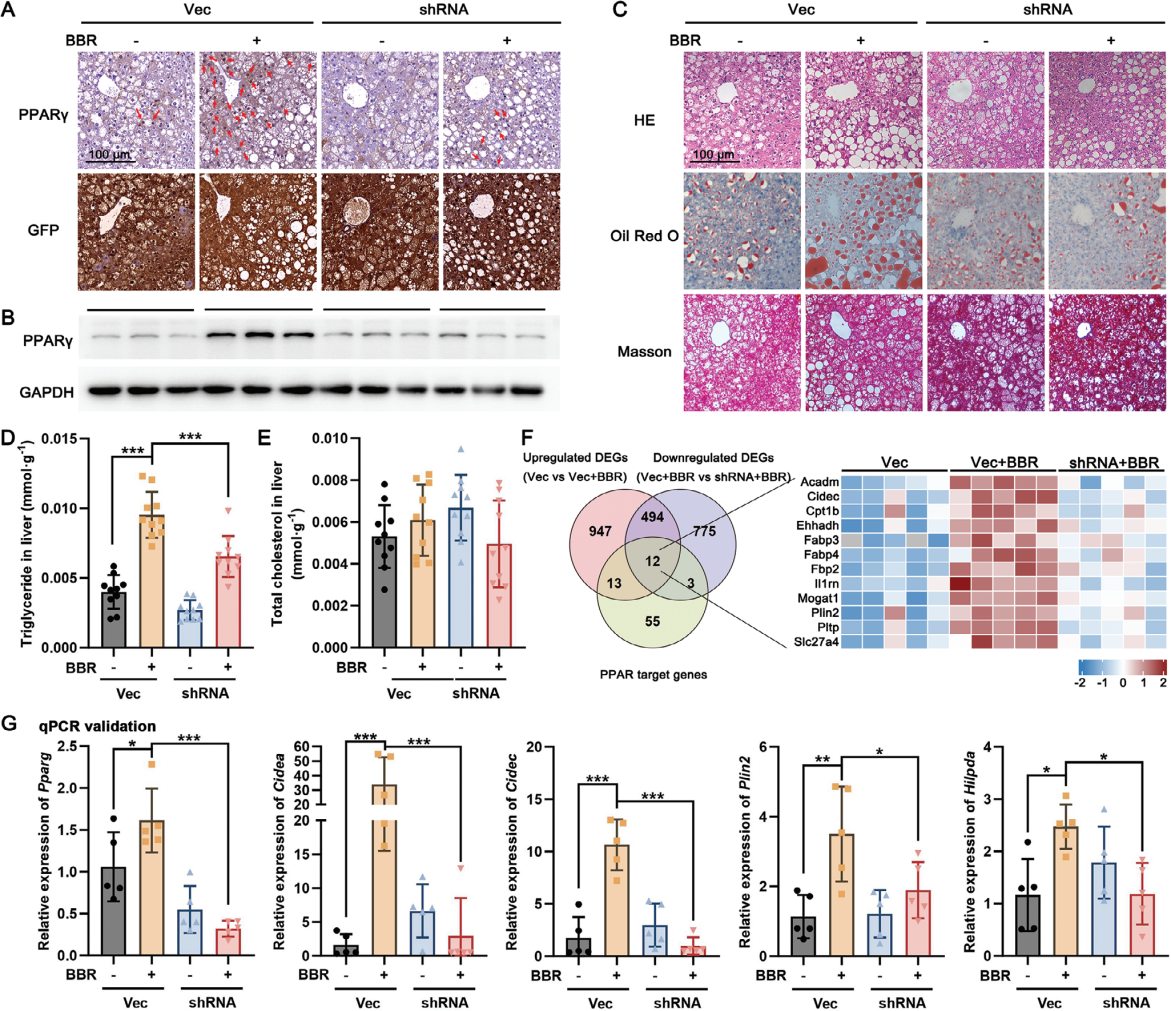
Knockdown of PPARγ restores benzbromarone‐induced aggravation of hepatic steatosis in db/db mice. A) The expression and tissue localization of PPARγ and green fluorescent protein (GFP) were determined in situ by immunohistochemistry, scale bar = 100 µm. B) The expression of PPARγ in livers from each group was detected by western blotting, using GAPDH as an internal control; the blots were repeated 3 times. C) Representative images of hematoxylin‐eosin (HE), Oil Red O, and Masson staining in each group, scale bar = 100 µm. D,E) The levels of triglycerides and total cholesterol (TC) in liver tissue were measured, ***p < 0.001 as indicated, *n* = 10. F) The Venn diagram and heatmap of DEGs in each group. G) The qPCR assay was utilized to assess the liver tissue expression levels of several representative DEGs, including Pparg, Cidea, Cidec, Plin2, and Hilpda. *p < 0.05, **p < 0.01, ***p < 0.001, *n* = 5.

Immunohistochemical staining and western blotting results validated the attenuation of BBR‐mediated PPARγ expression and nuclear translocation within the hepatocytes of db/db mice subjected to PPARγ‐shRNA treatment (**Figure** **6**A,B). The morphological results, as demonstrated by H&E, Masson's trichrome, and Oil Red O staining, conclusively illustrated that targeted knockdown of PPARγ in the liver significantly mitigated the exacerbation of ballooning degeneration and lipid accumulation within hepatocytes of BBR‐treated db/db mice (Figure 6C). The collagen deposition in BBR‐treated db/db mice was also found to be less altered, indicating that the process of transitioning from hepatic steatosis to fibrosis did not occur within 4‐week treatment period (Figure 6C). Consistent with the morphological findings, the elevation of liver triglyceride levels induced by BBR was markedly attenuated by PPARγ knockdown (Figure 6D), while the total cholesterol levels in the liver displayed minimal variations among the four groups (Figure 6E). Furthermore, the upregulation of various PPARγ target genes related to lipid metabolism, as initially elicited by BBR, was conspicuously mitigated by the hepatic‐specific knockdown of PPARγ in db/db mice (Figure 6F,G).

### 2.7 The PPARγ‐Selective Antagonist GW9662 Alleviates Benzbromarone‐Induced Aggravation of Hepatic Steatosis and Liver Injury in db/db Mice

**Figure 7:**
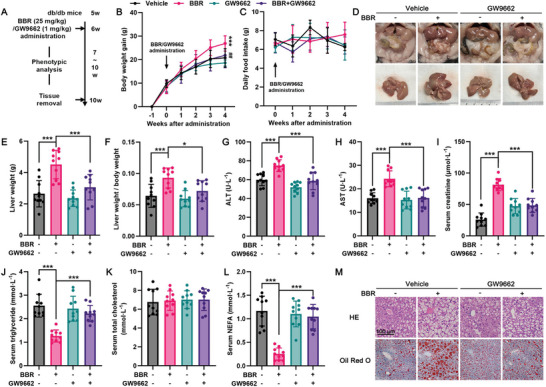
The PPARγ‐selective antagonist GW9662 alleviates benzbromarone‐induced aggravation of hepatic steatosis and liver injury in db/db mice. A) Flow scheme of GW9662/BBR co‐treatment in db/db mice. B) Body weight gain, C) daily food intake in different groups. D) Representative images of the livers and other abdominal organs are shown. E) Liver weights and F) liver weights normalized by body weight in each group. G) Serum ALT, H) AST, I) creatinine, J) triglyceride, K) total cholesterol, and L) NEFA levels. *p < 0.05, ***p < 0.001, *n* = 10. M) Representative image of HE and Oil Red O staining in each group, scale bar = 100 µm.

The involvement of PPARγ agonism in exacerbating hepatic steatosis and liver damage triggered by BBR in db/db mice was confirmed using a pharmacologically targeted strategy involving the administration of GW9662, a selective PPARγ antagonist (**Figure** **7**A). A 4‐week concurrent treatment with GW9662 markedly mitigated the BBR‐mediated exacerbation of hepatic steatosis and liver injury in db/db mice as indicated by a substantial reduction in liver weight and size (Figure 7B–F), as well as a notable decline in serum levels of ALT, AST, and creatinine (Figure 7G–I). In addition, the levels of serum triglyceride, total cholesterol, and NEFA (Figure 7J–L), as well as the glucose profiles (Figure S2, Supporting Information), were also similar to those observed following the genetic knockdown of PPARγ in the livers of db/db mice, thereby reinforcing the crucial role played by PPARγ in the detrimental effects of BBR.

### 2.8 Slow Metabolism of Benzbromarone Enhances its Hepatic Steatogenic Effect via Amplification of PPARγ Agonism in Obese Mice

**Figure 8:**
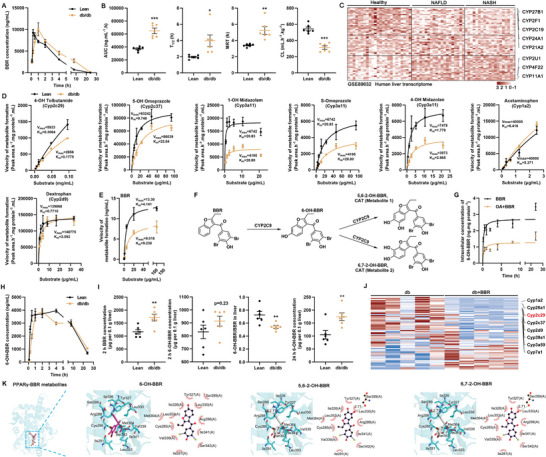
Slow metabolism of benzbromarone enhances its hepatic steatogenic effect by amplification of PPARγ agonism in obese mice. A) Plots of the plasma pharmacokinetic profiles of BBR in different groups, *n* = 6 per time point. B) Comparison of the pharmacokinetics parameters, area under the curve (AUC), half‐life (T_1/2_), mean residence time (MRT), and clearance (CL) for BBR, *n* = 6. C) Heatmap depicting the expression of CYP450 enzymes in the DEGs derived from RNA sequencing data of the liver tissue from healthy humans (*n* = 24), patients with NAFLD (*n* = 20) or NASH (*n* = 19), sourced from the publicly available dataset GSE89632. D) Kinetic profiles of CYP450s substrate metabolism after incubation using pooled liver S9 fractions from db/db mice and their lean littermates, *n* = 5. E) Kinetic profile of BBR after incubation with liver S9 fractions. F) Proposed metabolic pathway of BBR. G) Quantification of intracellular 6‐OH‐BBR concentrations in primary hepatocytes after incubation with BBR for the different times in the presence/absence of OA, *n* = 6. H) Plots of the plasma pharmacokinetic profiles of 6‐OH‐BBR in different groups, *n* = 6 per time point. I) Kinetic profiles of 6‐OH‐BBR production in db/db mice and their lean littermates, *p < 0.05, **p < 0.01, ***p < 0.001, *n* = 6. J) Heatmap of the expression of CYP450 enzymes in liver DEGs from BBR‐treated db/db mice compared to vehicle groups. K) In silico docking of BBR, 6‐hydroxy‐BBR (6‐OH‐BBR), 5,6‐dihydroxy‐BBR (5,6‐2‐OH‐BBR), and 6,7‐dihydroxy‐BBR (6,7‐2‐OH‐BBR) with PPARγ.

Unlike the case for the lean C57/BL6 mice, the plasma concentration‐time profile of BBR in db/db mice displayed significant differences, with a 1.75‐fold increase in the area under the curve (AUC), a 2.03‐fold increase in plasma half‐life (T_1/2_), a 1.54‐fold increase in mean residence time (MRT), and a 42.5% decrease in clearance rate (CL), indicative of a pronounced accumulation of plasma BBR in obese mice (**Figure** **8**A,B). Given that hepatic metabolic change is a crucial factor contributing to drug accumulation and that multiple CYP450 enzymes are downregulated in patients with NAFLD and NASH (Figure 8C), we proposed that hepatic steatosis leads to a slow metabolism of BBR, resulting in BBR accumulation.

To test this hypothesis, we first verified the reduction in the activities of hepatic CYP450 enzymes, particularly Cyp2c29, Cyp2c37, and Cyp3a11, in db/db mice through enzymatic kinetic experiments (Figure 8D). BBR undergoes primary metabolism via hepatic CYP2C9 (analogous to Cyp2c29 in mice) to yield 6‐hydroxy‐benzbromarone (6‐OH‐BBR) and subsequent hydroxylated derivatives (Figure 8F). Consistent with the decreased expression and activity of liver Cyp2c29 in hepatic steatosis, the metabolism of BBR catalyzed by liver S9 fractions isolated from db/db mice exhibited a marked reduction compared to those from lean mice (Figure 8E). This finding was further validated in primary mouse hepatocytes incubated with OA (Figure 8G). The plasma concentration‐time profile of 6‐OH‐BBR suggested delayed production in db/db mice compared to their lean littermates (Figure 8H). At 2 h post‐administration, BBR concentrations were notably higher in db/db mouse livers with a lower metabolite‐to‐parent drug ratio. By 24 h, BBR was undetectable in the liver tissues of both groups and yet the db/db mice exhibited significantly higher concentrations of 6‐OH‐BBR compared to their lean littermates (Figure 8I). These findings collectively indicated an impairment of BBR metabolism in db/db mouse livers that led to drug accumulation and delayed metabolite formation.

BBR treatment also reduced the expression of multiple CYP450 enzymes, especially Cyp2c29, in the livers of db/db mice (Figure 8J). This reduction may further exacerbate the retention of BBR in hepatocytes. Our study also revealed that the CYP2C9 metabolites of BBR, including 6‐OH‐BBR, 5,6‐dihydroxy‐benzbromarone (5,6‐2‐OH‐BBR), and 6,7‐dihydroxy‐benzbromarone (6,7‐2‐OH‐BBR), shared similar binding sites with BBR on PPARγ, as evidenced by in silico docking experiments (Figure 8K). The binding poses of 6‐OH‐BBR and 5,6‐2‐OH‐BBR are nearly identical to that of BBR, which involves binding sites located deep within the hydrophobic pocket and the formation of hydrophobic interactions with residues Try327, Leu330, Arg288, Ile341, Ser342, Ile281, Val339, Cys285, Leu353, Met364, and Ile326, suggesting potential PPARγ agonism. Both 5,6‐2‐OH‐BBR (−8.23 kcal·mol^−1^) and 6‐OH‐BBR (−7.76 kcal·mol^−1^) had better docking scores than BBR (−7.58 kcal·mol^−1^), whereas 6,7‐2‐OH‐BBR, which binds at a shallower position in the hydrophobic pocket, showed a slightly lower docking score than BBR (Figure 8K).

## 3 Discussion

Globally, the prevalence of hyperuricemia and gout has been escalating annually, and yet the clinical options for urate‐lowering therapy remain constrained.^[^ ^12^ ^]^ Among the three established and approved urate‐lowering medications utilized in clinical practice, allopurinol may cause drug‐induced hypersensitivity syndrome (DiHS) in patients who possess the HLA‐B*58:01 allele,^[^ ^13^ ^]^ whereas the novel xanthine oxidase (XO) inhibitor febuxostat is not recommended for the management of asymptomatic hyperuricemia.^[^ ^14^ ^]^ Therefore, despite its withdrawal from European and American markets due to hepatotoxicity concerns, BBR, a URAT1 inhibitor, remains widely prescribed in various countries as a urate‐lowering agent.^[^ ^15^ ^]^ Consequently, elucidating its toxicological mechanisms remains an important global issue.

Most prior investigations have implicated mitochondrial toxicity as the primary driver of BBR‐induced liver injury. However, our recent findings introduced a novel perspective, suggesting that BBR primarily exacerbates hepatic steatosis, thereby precipitating severe DILI specifically in obese individuals, while exhibiting a lesser impact on lean subjects.^[^ ^8^ ^]^ Within the limited experimental period, no transition from hepatic steatosis to fibrosis was observed in BBR‐treated db/db mice. However, it can be reasonably hypothesized that severe liver injury and fibrosis may occur with long‐term BBR administration. BBR shows a modest dual activation of PPARα and PPARγ.^[^ ^9^ ^]^ However, by leveraging multi‐omics, in silico, genetic, and pharmacological analyses in the present study, we have confirmed that hepatic lipid accumulation mediated by the agonistic activity of PPARγ, but not PPARα, is the primary contributor to BBR‐induced obesity‐specific DILI, in agreement with observations made with selective PPARγ agonists, such as rosiglitazone or pioglitazone.^[^ ^16^ ^]^ Despite numerous clinical and foundational studies highlighting the positive influence of the PPARγ agonist TZDs in mitigating non‐alcoholic fatty liver disease (NAFLD) and non‐alcoholic steatohepatitis (NASH),^[^ ^17^ ^]^ subsequent studies have cautioned that TZDs can, in some cases, exacerbate liver triglyceride accumulation and hepatic steatosis due to their PPARγ agonistic activities.^[^ ^18^ ^]^ Thus, the BBR‐induced obesity‐specific DILI is plausibly a result of its agonistic activity on PPARγ. However, one crucial question remains unanswered, necessitating further inquiry: How can BBR, a relatively modest PPARγ agonist, elicit a more marked influence on hepatic lipid accumulation compared to selective PPARγ agonists with much higher activity?

The answer to this question may lie in the growing evidence underscoring the significant impact of obesity and NAFLD in elevating the risk of DILI. Obese individuals, when compared to healthy lean individuals, exhibit notable alterations in various pharmacokinetic parameters, including intestinal absorption, plasma protein binding, hepatic extraction ratio, hepatic blood flow, portosystemic shunting, biliary excretion, enterohepatic circulation, and renal clearance.^[^ ^19^ ^]^ These alterations can lead to increased drug bioavailability and accumulation, thereby predisposing obese individuals to drug toxicity even when the drugs are administered at standard therapeutic doses.^[^ ^19a^ ^]^

Our exploration of a public transcriptomic dataset also illustrated a profound perturbation in the expression patterns of CYP450 enzymes among patients with NAFLD or NASH. BBR is metabolized in the liver by CYP2C9 (analogous to Cyp2c29 in mice) to produce 6‐hydroxy‐BBR and other hydroxylated metabolites.^[^ ^20^ ^]^ Our study highlighted profound perturbations in the expression and activity of CYP450 enzymes associated with hepatic steatosis, as supported by comprehensive in vivo and in vitro experimental evidence and clinical transcriptomic data. BBR is primarily metabolized and eliminated by liver CYP2C9 (analogous to Cyp2c29 in mice).^[^ ^20^ ^]^ Our in vivo pharmacokinetic profile assays, liver S9 enzyme kinetics, and primary hepatocyte studies revealed that hepatic steatosis‐induced reduction in CYP2C9 substantially decreased BBR clearance, leading to drug accumulation and delayed metabolite formation. Notably, we found that the BBR metabolites (6‐OH‐BBR and other hydroxylated derivatives), like the parent compound, retain the ability to engage in binding interactions with PPARγ. This suggests that the prolonged accumulation of BBR and its metabolites due to impaired hepatic metabolism in hepatic steatosis results in sustained activation of PPARγ, thereby amplifying the PPARγ agonistic activity of administered BBR. Our study further demonstrates that BBR activation of PPARγ leads to increased hepatic lipid accumulation and injury, along with a more pronounced reduction in CYP450 expression in hepatic steatosis. This creates a cycle that culminates in BBR, causing obesity‐specific DILI.

The findings presented here have significant implications: Despite its hepatic toxicity, the robust uric acid‐lowering efficacy of BBR has ignited a surge in research aimed at discovering novel URAT1 inhibitors based on the BBR chemical backbone.^[^ ^5^ ^]^ Recent endeavors have strategically focused on mitigating mitochondrial toxicity through the avoidance of benzoquinone in the structure of these novel URAT1 inhibitors,^[^ ^5a^ ^]^ informed by a prior understanding of the toxicological mechanisms of BBR. However, the revelation of this obesity‐specific mechanism of hepatic toxicity in our study underscores a pivotal new consideration in the pursuit of designing innovative URAT1 inhibitors derived from the structural scaffold of the necessity of ascertaining their potential PPARγ agonistic activities. Our molecular docking studies have shown that the core benzofuran structure of BBR can bind to PPARγ at its active ligand‐binding pocket to mirror the interaction between PPARγ and its well‐known agonist, rosiglitazone. This suggests that the benzofuran structure plays a pivotal role in the CYP2C9‐mediated metabolism and PPARγ agonistic activity of benzbromarone.

Correspondingly, other drugs containing a similar benzofuran structure, such as amiodarone and benzarone, have also been reported to cause hepatic lipid infiltration and hepatotoxicity.^[^ ^6b^ ^]^ To investigate this further, we utilized in silico docking with PPARγ to assess the binding activity of several novel URAT1 inhibitors, designed based on the structural scaffold of BBR. Our findings revealed PPARγ agonistic activity comparable to that of BBR itself (Table S4, Supporting Information). Therefore, avoiding PPARγ agonistic activity may be equally critical as preventing mitochondrial toxicity for mitigating hepatotoxicity when pursuing novel URAT1 inhibitors derived from the structural scaffold of BBR.

Another ramification of the present study, given the escalating global obesity epidemic, is that the findings underscore the pressing requirement to address obesity‐specific DILI in the realm of drug discovery. While reduced mitochondrial functions or heightened oxidative stress may offer plausible explanations for the exacerbated obesity‐specific DILI observed with certain drugs, acknowledging the emergence of novel mechanisms, such as the additional hepatic activation of PPARγ by TZDs or BBR, is equally crucial. Our recent findings draw concerns regarding the case of Muparfostat, an advanced heparan sulfate mimetic currently undergoing anti‐tumor Phase III clinical trials. Muparfostat exhibits additional binding affinity toward lipoprotein lipase, thereby causing severe hyperlipidemia and ultimately exacerbating hepatic steatosis and liver damage, particularly in obese individuals.^[^ ^21^ ^]^

This discovery significantly broadens our understanding of the complexities underlying obesity‐specific DILI. Notably, variations in pharmacokinetic profiles between obese individuals and those with NAFLD have been documented extensively, as evidenced by prior studies.^[^ ^22^ ^]^ BBR, with its heightened retention in the liver, potentiates an additional pharmacological effect—PPARγ agonism. This observation emphasizes the criticality of acknowledging the potential effects on hepatic PPARγ activity during drug development, especially the development of medications anticipated for use in obese populations, given the potential for unforeseen liver damage. Thus, the present findings further highlight the need for a heightened awareness and proactive approach to mitigating obesity‐related DILI risks in the drug discovery process.

Notably, given the crucial role of PPARγ in regulating glucose and lipid metabolism in peripheral organs such as adipose tissue and skeletal muscle, as well as its involvement in immune modulation, global pharmacological inhibition of PPARγ, like GW9662, may lead to some off‐target effects beyond the liver. Therefore, for the high‐risk DILI population of obese individuals, we do not recommend targeting PPARγ as a potential clinical strategy for intervention. Instead, our findings provide evidence that necessitates a reevaluation of the therapeutic potential of BBR in non‐obese individuals, suggesting that this patient cohort may derive benefit from BBR treatment with a reduced risk of DILI. Conversely, for obese individuals, we recommend avoiding the use of BBR and instead opting for alternative urate‐lowering therapies, such as allopurinol and febuxostat, which should be managed similarly to other medications that have the potential to induce DILI.

## 4 Experimental Section

### Animal Model

The animal experiments were conducted with approval from the Animal Care and Ethics Committee of Nanjing Medical University (2 012 023). C57/BL6 mice, Lepr db/db mice, and their lean littermates (all male and 5 weeks old) were purchased from GemPharmatech Co., Ltd. To knock down the expression of PPARγ in the liver, the 4‐in‐1 shRNA (Table S1, Supporting Information) target mouse Pparg (NM_0 011 27330) was introduced into an adeno‐associated virus 8 (AAV8) vector obtained from WZ biosciences (Jinan, China), with an AAV8 vector containing non‐targeting shRNA used as control. The AAV8‐PPARγ‐shRNA and AAV8‐vector were injected into 5‐week‐old mice through the tail vein at 1 × 10^12^ vector genome (VG) per mouse. Benzbromarone (BBR, HY‐B1135, MedChemExpress, Shanghai, China) was dissolved in 0.5% sodium carboxymethyl cellulose (CMC‐Na), stored at 4 °C, and administered via gavage at a dose of 25 mg·kg^−1^ once daily for 4 weeks. The vehicle group received CMC‐Na only. For pharmacological inhibition of PPARγ, GW9662 (HY‐16578, MedChemExpress) was intraperitoneally injected at 1 mg·kg^−1^ body weight with/without BBR. The fasting (6 h) plasma glucose (FPG), body weight, and food consumption were assessed weekly. At the end of administration, tissues and blood samples were collected following a 6 h fasting period.

### Glucose Tolerance, Pyruvate Tolerance, and Insulin Tolerance Tests

The glucose tolerance tests (GTT), pyruvate tolerance tests (PTT), and insulin tolerance tests (ITT) were performed at the end of administration as previously described.^[^ ^21^ ^]^ The area under the curve (AUC) or the rate of change in blood glucose from 0 to 60 min (Δglucose0‐60) was computed to assess and compare the GTT, PTT, and ITT results across the various groups.

### Serum and Liver Biochemical Analyses

Blood samples were collected and centrifuged at 1000 g for 10 min to extract clear serum for biochemical analysis. Serum concentrations of alanine aminotransferase (ALT), aspartate aminotransferase (AST), creatinine, triglycerides, total cholesterol, non‐esterified fatty acids (NEFAs), very low‐density lipoprotein (VLDL), low‐density lipoprotein (LDL), and high‐density lipoproteins (HDL) were measured using commercial assay kits (Jiancheng Bio Research Institute, Nanjing, China). Serum insulin levels were measured using an ELISA kit (MZ‐060, Ezassay Co., Ltd., Shenzhen, China) following the manufacturer's procedure.

Lipid concentrations in liver tissues were measured using a chloroform/methanol‐based method, as described previously.^[^ ^23^ ^]^ Triglyceride and total cholesterol concentrations were measured using commercial assay kits (Jiancheng) and normalized to liver weight.

### Tissue Harvesting and Histology

Mice were euthanized, and different tissues were removed. Histological staining was performed as previously described.^[^ ^21^ ^]^ Briefly, for hematoxylin/eosin (H&E) and Masson staining, tissues were fixed in 4% paraformaldehyde, embedded in paraffin, and sectioned into 5 µm tissue slices. For Oil Red O staining, tissues were frozen at −80 °C, embedded in optimal cutting temperature compound (OCT), and cut into 10 µm frozen sections.

### Immunohistochemical Staining and Western Blotting Analysis

Immunohistochemical staining and western blotting were performed as previously described.^[^ ^24^ ^]^ Briefly, paraffin sections were dewaxed, rehydrated, and incubated with anti‐PPARγ (#2435, Cell Signaling Technology, Danvers, MA, USA), or anti‐GFP (#50430‐2‐AP, Proteintech, Chicago, IL, USA) primary antibodies.

For western blotting analysis, the anti‐PPARγ (#2435, Cell Signaling Technology) primary antibody was used, and anti‐GAPDH (ab9485, Abcam, Cambridge, MA, USA) was used as an internal control. All blots were repeated at least three times.

### Isolation of Primary Hepatocytes and Oleic Acid Treatment

Primary hepatocytes were isolated from the livers of 6‐week‐old male C57BL/6J mice as previously described.^[^ ^25^ ^]^ Briefly, the liver was thoroughly digested by injection of type IV collagenase (C4‐BIOC, Sigma‐Aldrich, Louis, MO, USA) via the portal vein. The perfused liver was excised, dispersed, and filtered through a 70 µm filter, and the digestion was terminated by adding cold Dulbecco's modified Eagle's medium (DMEM, #11 960 044, Gibco, Grand Island, NY, USA). The cell suspension was centrifuged at 4 °C at 50 g for 5 min to remove the supernatant and debris. An equal volume of DMEM and Percoll (CAS 65455‐52‐9, Sigma‐Aldrich) was then mixed thoroughly and centrifuged at 50 g for 15 min at 4 °C. The viable cell layer at the bottom was collected and further centrifuged in DMEM to prepare the cell seeding plate. Cells were seeded in a 6‐well plate at a density of 1 × 10^6^ cells per well and cultured in DMEM medium supplemented with 10% fetal bovine serum (FBS, Gibco) at 37 °C in a 5% CO_2_ incubator.

To establish an in vitro model of hepatic steatosis, hepatocytes were treated with 0.2 mm oleic acid (OA, O7501, Sigma‐Aldrich) for 16 h. OA was pre‐incubated with 10% bovine serum albumin (BSA, Sigma‐Aldrich) (1:4) for 1 h and then added to the cell culture medium along with different compounds.

### Cell Viability Assay

Primary hepatocytes were seeded in 96‐well plates and incubated with different doses of BBR for 24 h. The cell viability was measured using a cell counting kit‐8 (CCK8) (A311‐01/02, Vazyme Biotech Co., Ltd.), and the cytotoxicity was calculated as the 50% inhibitory concentration (IC50).

### Measurement of Intracellular Triglyceride Content and BODIPY Staining

Primary hepatocytes were washed three times with phosphate‐buffered saline (PBS) and then subjected to three repeated freeze‐thaw cycles. The released intracellular triglyceride content was then measured using a commercial assay kit (Jiancheng). Protein content was used for normalization.

The lipid droplets in the hepatocytes in 6‐well plates were visualized by first washing the cells and then immersing them in a serum‐free medium. Subsequently, 10 mm BODIPY 493/503 (Sigma‐Aldrich) was added to the medium, and the cells were incubated for 20 min for staining, followed by imaging with confocal microscopy (Olympus, Tokyo, Japan).

### RNA Extraction and Real‐Time PCR

Tissues or cells were lysed using TRIzol (#1 218 355, Invitrogen, Thermo Fisher Scientific, MA, USA) for subsequent RNA extraction through a series of steps involving chloroform extraction and isopropanol and ethanol precipitation. Reverse transcription and cDNA synthesis were performed using a reverse transcription and cDNA synthesis kit (#639 506, Takara Bio Inc., Kusatsu, Shiga, Japan) to synthesize complementary DNA (cDNA) from 0.5 mg of RNA. Quantitative real‐time PCR analysis was conducted utilizing SYBR Green pre‐mix (Q111‐02, Vazyme Biotech Co., Ltd, Nanjing, China) and subsequently analyzed employing the Roche real‐time PCR system. The mRNA expression levels were calculated and quantified utilizing the relative standard curve method and subsequently normalized to the expression of the housekeeping gene Gapdh. The primer sequences utilized for qPCR analysis are listed in Table S2 (Supporting Information).

### RNA Sequencing and Lipidomic Analysis

Total RNA was extracted from cultured hepatocytes or liver tissues to obtain the cDNA libraries, and RNA sequencing was performed on the Illumina sequencing platform by Genedenovo Biotechnology Co., Ltd. (Guangzhou, China). All RNA sequencing data have been deposited in the GEO database with the accession number GSE274374 and GSE274748.

For lipidomic analysis, lipids were extracted from cultured hepatocytes using the improved Bligh/Dyer extraction method, and the lipidomic analyses were conducted at LipidALL Technologies (Beijing, China) using a Shimadzu Nexera 20AD‐HPLC/ExionLC‐AD coupled with Sciex QTRAP 6500 PLUS, as reported previously.^[^ ^26^ ^]^ The concentrations of triglycerides (TAG), diglycerides (DAG), cholesterol esters (CE), free fatty acids (FFA), acylcarnitine, and acyl‐coenzyme A were normalized to the protein content.

### Molecular Docking

Glide (Schrödinger version 2020) was used for molecular docking. Three complex crystal structures of human PPARα, namely (PDB ID: 6kba), PPARβ/δ (PDB ID: 3tkm), and PPARγ (PDB ID: 5ycp), were obtained from the Protein Data Bank. The protein Preparation Wizard tool was utilized to refine and optimize the three crystal structures through the OPLS3 force field. The 3D structures of the compounds were generated using the LigPrep tool at pH 7.4 with EPIK. Docking grids were created with default parameters, while co‐crystallized ligands were used to determine the center of the grid box. Protein‐ligand docking was performed in the standard precision mode. The compounds were docked to PPARα, PPARβ/δ, and PPARγ. The binding free energies were calculated using the MM/GBSA method. The conformations were ranked according to the results, and the best conformation was selected for further analysis.

### MicroScale Thermophoresis (MST) Assay

The direct binding activity of rosiglitazone to recombinant human PPARγ protein (Sino Biotechnology, Beijing, China) in the presence/absence of BBR was detected using a MonolithTM RED‐NHS kit (Cat# MO‐L011, NanoTemper, South San Francisco, CA, USA) in the MST Capillaries Monolith NT.115 (NanoTemper) following the manufacturer's procedure.

### Pharmacokinetic Profile of Benzbromarone in vivo

To investigate the metabolism of BBR in obese individuals, an identical single dose of BBR (20 mg·kg^−1^, i.g.) was administered to both db/db mice and their lean littermates, and compared the pharmacokinetic behavior of BBR and its major metabolite 6‐hydroxy‐BBR (6‐OH‐BBR) in these two mouse models. Briefly, blood samples (50 µL) were collected at different time points post‐drug administration (no more than three collections per mouse), centrifuged at 4 °C and 8000 g for 5 min, and the upper plasma layer was collected. At 2 and 24 h post‐dosing, the mice were sacrificed, and liver tissues were collected for cryopreservation and prepared as homogenates (W/V: 0.1 g·mL^−1^) for drug concentration determinations. The concentrations of BBR and 6‐OH‐BBR in plasma and liver homogenates were determined by liquid chromatography‐tandem mass spectrometry (LC‐MS/MS).

### The Metabolism of Benzbromarone in Cultured Mouse Primary Hepatocytes in the Presence/Absence of Oleic Acid

The in vitro metabolic profile of BBR was determined in cultured murine primary hepatocytes in the presence/absence of oleic acid. Briefly, primary hepatocytes were treated with 0.2 mm oleic acid for 16 h to establish an in vitro hepatic steatosis model. Subsequently, the cells were exposed to 15 µm BBR at 37 °C for the requisite period. For post‐treatment, cells were washed with ice‐cold PBS, collected, and sonicated to prepare a homogenate. The concentrations of BBR and its metabolite, 6‐OH‐BBR, within the homogenate, were quantified by LC‐MS/MS and normalized to the protein content of the corresponding samples.

### Enzyme Kinetic Study

Kinetic studies for multiple cytochrome P450 isoforms were conducted using a graded series of substrate concentrations with liver S9 fractions from db/db mice or their lean littermates: acetaminophen (0.078–2.5 µg·mL^−1^) for Cyp1a2; tolbutamide (3.0–96.0 µg·mL^−1^) for Cyp2c29; omeprazole (2.91–93.15 µg·mL^−1^) for Cyp2c37 and Cyp3a11; dextrophan (1.04–33.2 µg·mL^−1^) for Cyp2d9; and midazolam (0.66–21.0 µg·mL^−1^) for Cyp3a11. The incubations were carried out for 2 h at 37 °C in a 100 µL reaction volume containing the pooled liver S9 fraction (7.5 mg·mL^−1^) and an NADPH‐regenerating system consisting of MgCl_2_ (40 mm), glucose‐6‐phosphate (40 µm), NADP^+^ (8 µm), and glucose 6‐phosphate dehydrogenase (4 U·mL^−1^). The reactions were terminated by the addition of 300 µL of ice‐cold acetonitrile containing the internal standard (chlorophenylalanine), followed by rigorous mixing and centrifugation. The supernatants were subsequently analyzed by LC‐MS/MS, employing methodologies detailed in previous publications.^[^ ^27^ ^]^

### LC‐MS/MS‐Based Quantitative Analysis of Benzbromarone and its Metabolites

Briefly, proteins in the samples were precipitated with three volumes of methanol containing an internal standard (chlorophenylalanine). After centrifugation at 30 000 g for 10 min at 4 °C, the supernatant was injected into the LC‐MS/MS system for analysis. Chromatographic separation was conducted using a ZORBAX 300SB C18 column (100 × 2.1 mm, 3.5 µm, Agilent, CA, USA) at 40 °C on a Shimadzu HPLC system. The mobile phase consisted of solvent A (0.1% aqueous formic acid) and solvent B (methanol) at a flow rate of 0.3 mL·min^−1^, and the following 12 min gradient was used: 0 min, 10% B; 1.5 min, 10% B; 2.5 min, 90% B; 8.5 min, 90% B; 9 min, 10% B; 12 min, 10% B. The mass spectrometer was operated in negative electrospray ionization (ESI) mode, and the following multiple reaction monitoring (MRM) parameters were set: declustering potential set at −50 V for BBR, ‐20 V for 6‐OH‐BBR, and −55 V for IS; collision energy set at −42 eV for BBR, −40 eV for 6‐OH‐BBR, and −18 eV for IS; and MRM transition set as m/z 423.1→250.8 for BBR, 439.1→250.8 for 6‐OH‐BBR, and 198.0→180.4 for IS.

### GEO Data Retrieval and Bioinformatic Analysis

The gene expression profile datasets GSE27183,^[^ ^28^ ^]^ GSE17250,^[^ ^29^ ^]^ GSE27948 and GSE89632,^[^ ^30^ ^]^ were downloaded from the GEO database (https://www.ncbi.nlm.nih.gov/geo/). R packages, Limma,^[^ ^31^ ^]^ and edgeR,^[^ ^32^ ^]^ were used to analyze the differentially expressed genes (DEGs) between two groups. Genes with statistically significant differences (*p* < 0.05) were identified as DEGs. The R package ClusterProfiler,^[^ ^33^ ^]^ was used for the Kyoto Encyclopedia of Genes and Genomes (KEGG) and Gene Set Enrichment Analysis (GSEA) pathway enrichment analyses. Terms with statistically significant differences (*p* < 0.05) were identified as significantly enriched terms. Visualization was performed using the ggplot2, pheatmap, and Gseavis R packages.

### Statistical Analysis

All values were presented as means ± standard deviation (SD). The sample size (n) was shown in the figures or legends, where applicable. GraphPad Prism (Version 8.0, USA) software was used to analyze the data. The two‐tailed unpaired Student's *t*‐test was used to compare significant differences between the two groups, while one‐way ANOVA followed by the Turkey test was used for three or more groups. A value of *p* < 0.05 was considered statistically significant.

## Conflict of Interest

The authors declare no conflict of interest.

## Author Contributions

G.L., Y.H., H.Z., and Z.P. contributed equally to this work. Z.X., J.L., and P.S. designed and supervised the study. G.L., Y.H., H.Z., Z.P., X.S., J.Z., K.X., M.L., X.Z., and K.L. performed the experiments. G.L., Y.H., and P.S. analyzed and interpreted the data. G.L., Y.H., H.Z., H.W., Z.X., J.L., and P.S. drafted and revised the manuscript. Z.X., J.L., and P.S. approved the manuscript for submission.

## Supporting information



Supporting Information

## Data Availability

The data that support the findings of this study are available from the corresponding author upon reasonable request.
