# Supplementary material for: Slow Metabolism–Driven Amplification of Hepatic PPARγ Agonism Mediates Benzbromarone‐Induced Obesity‐Specific Liver Injury
Source: Adv Sci (Weinh). 2024 Nov 29;12(3):2409126. doi: 10.1002/advs.202409126 (PMC11744575; doi:10.1002/advs.202409126)
Supplement: Supplementary file 1 — Supporting Information [file ADVS-12-2409126-s001.docx]

Supporting Information

**Slow Metabolism–Driven Amplification of Hepatic PPARγ Agonism Mediates Benzbromarone-Induced Obesity-Specific Liver Injury**

*Guanting Li, Yourong Hu, Han Zhao, Ziyu Peng, Xin Shang, Jia Zhang, Kunxin Xie, Meiwei Li, Xiaohang Zhou, Qinyao Zhou, Kai Li, Fang Zhou, Heyao Wang, Zhijian Xu^*^, Jiali Liu^*^, Peng Sun^*^*

Supporting Display items, should be referred to as Figure S1, Table S1, etc., in the main text.


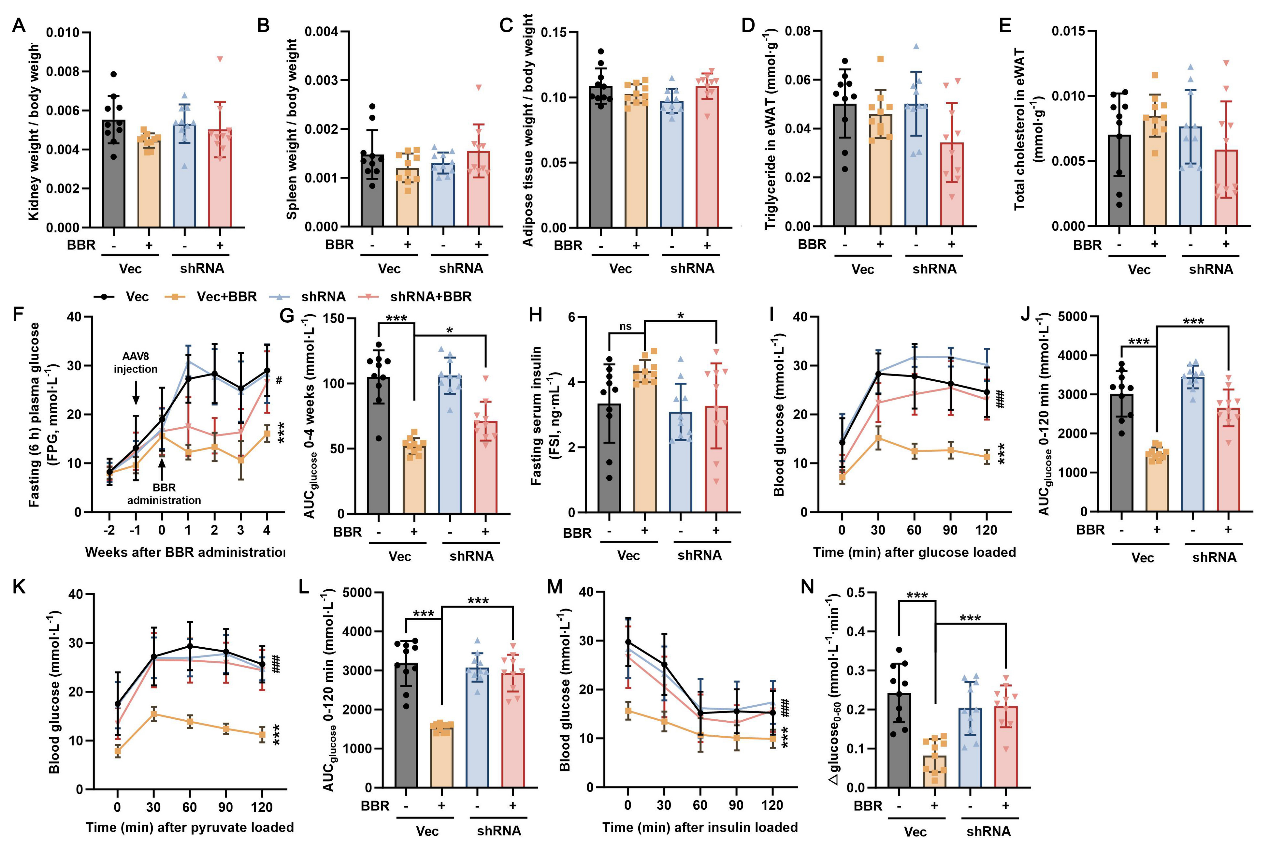


**Figure S1. The effect of hepatic knockdown of PPARγ on the glucose profiles of db/db mice following benzbromarone (BBR) administration.** (A-C) After treatment, the mice were euthanatized and tissues were removed. Kidney, spleen, and epididymal adipose (eWAT) tissues of each mouse were weighted and normalized by body weight. (D-E) The concentrations of triglyceride and total cholesterol in eWAT were measured and normalized by tissue weight. (F) Fasting plasma glucose (FPG) levels were recorded during treatment. (G) The area under the curve (AUC) of (F) was calculated. (H) After treatment, the fasting serum insulin (FSI) levels were measured. (I) Upon completion of the administration, the glucose tolerance test (GTT) curves along with their respective (J) AUC values, (K) the pyruvate tolerance test (PTT) curves and (L) AUC, (M) the insulin tolerance test (ITT) curves and (N) AUC were characterized and described. To calculate the statistical difference in (F), (I), (K), and (M), ***p<0.001 in Vec+BBR group compared to Vec group; #p<0.05, ###p<0.001 in shRNA+BBR group compared to Vec+BBR group, analyzed by two-way ANOVA analysis, n=10. For others, *p<0.05, ***p<0.001, ns=not significant, analyzed by one-way ANOVA analysis, as indicated, n=10.


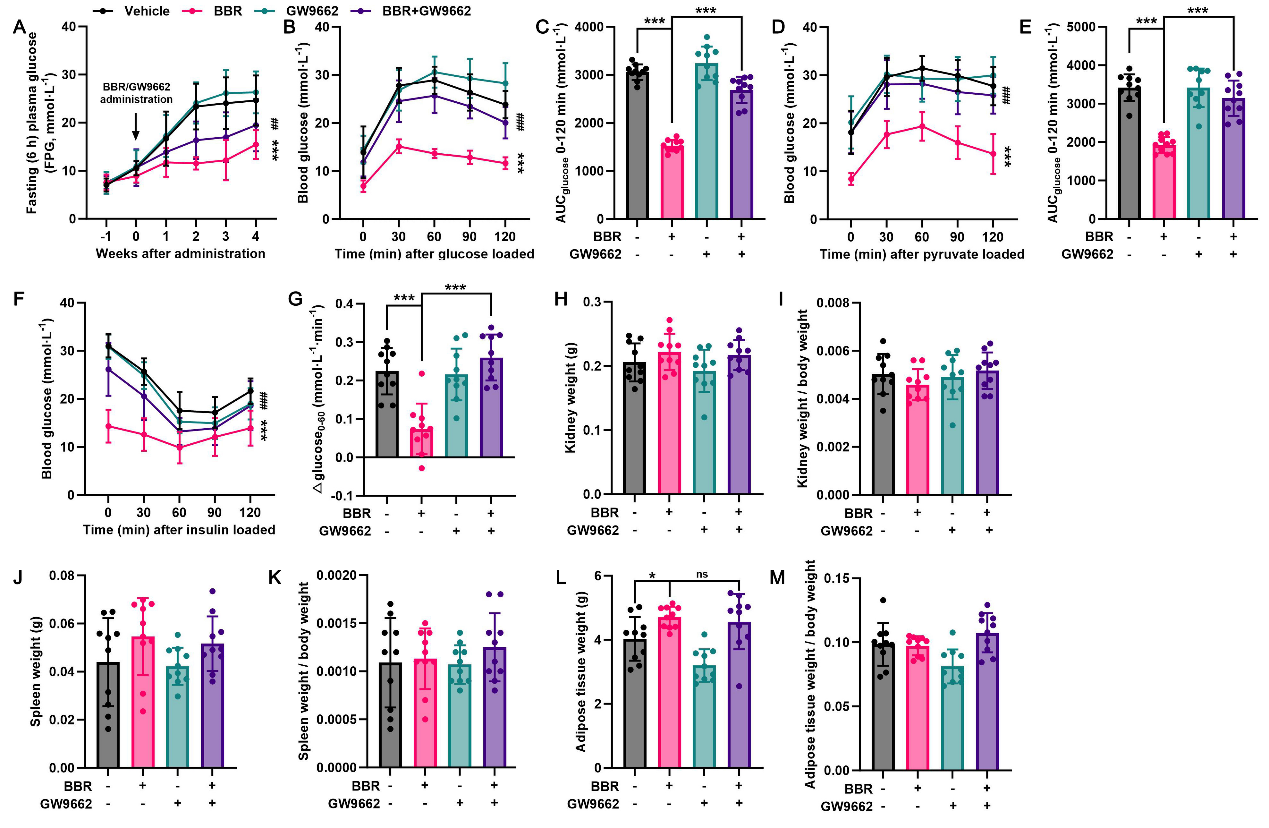


**Figure S2. The effect of benzbromarone (BBR) and/or GW9662 treatment on the glucose profiles of db/db mice.** (A) Fasting plasma glucose (FPG) levels were recorded during treatment. (B) Upon completion of the administration, the glucose tolerance test (GTT) curves along with their respective (C) AUC values, (D) the pyruvate tolerance test (PTT) curves and (E) AUC, (F) the insulin tolerance test (ITT) curves and (G) AUC were characterized and described. (H-M) After treatment, the mice were euthanatized and tissues were removed. Kidney, spleen, and epididymal adipose (eWAT) tissues of each mouse were weighted and normalized by body weight. To calculate the statistical difference in (A), (B), (D), and (F), ***p<0.001 in BBR group compared to Vehicle group; ##p<0.01, ###p<0.001 in BBR+GW9662 group compared to BBR group, analyzed by two-way ANOVA analysis, n=10. For others, *p<0.05, ***p<0.001, ns=not significant, analyzed by one-way ANOVA analysis, as indicated, n=10.


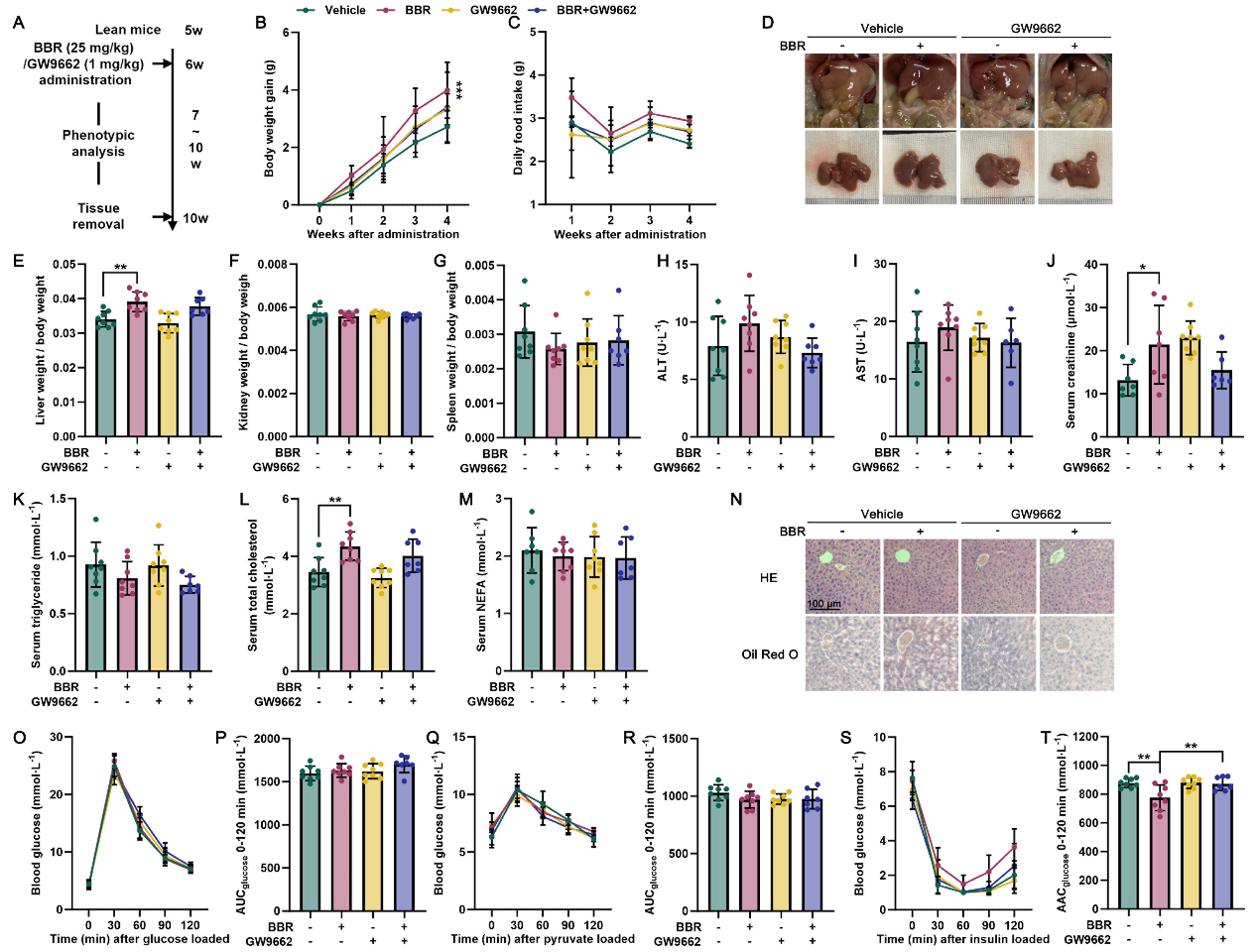


**Figure S3. The effect phenotypes of benzbromarone (BBR) and/or GW9662 treatment on lean mice.** (A) Flow scheme of BBR and/or GW9662 treatment in lean C57/BL6 mice. (B) Body weight gain, (C) daily food intake in different groups. ***p<0.001 in BBR group compared to Vehicle group, analyzed by two-way ANOVA analysis, n=8. (D) Representative images of the liver and other abdominal organs are shown. (E-G) The weights of the liver, kidney, and spleen of each animal were recorded and normalized by body weight. (H-M) The serum alanine aminotransferase (ALT), aspartate aminotransferase (AST), creatinine, triglyceride, total cholesterol, and non-esterified fatty acid (NEFA) concentrations were measured. (N) Representative images of hematoxylin-eosin (HE) and Oil Red O staining in each group, scale bar=100 μm. (O) Upon completion of the administration, the glucose tolerance test (GTT) curves along with their respective (P) AUC values, (Q) the pyruvate tolerance test (PTT) curves and (R) AUC, (S) the insulin tolerance test (ITT) curves and (T) AUC were characterized and described. *p<0.05, **p<0.01, as indicated, n=8.

**Table S1. PPARγ-shRNA sequences**

| PPARγ-shRNA sequences |  |
| --- | --- |
| shRNA1 | GCATCTCCACCTTATTATTCTTTCAAGAGAAGAATAATAAGGTGGAGATGCTTTTTT |
| shRNA2 | GGATGTCTCACAATGCCATCATTCAAGAGATGATGGCATTGTGAGACATCCTTTTTT |
| shRNA3 | GGAAAGACAACGGACAAATCATTCAAGAGATGATTTGTCCGTTGTCTTTCCTTTTTT |
| shRNA4 | GGAAGCCCTTTGGTGACTTTATTCAAGAGATAAAGTCACCAAAGGGCTTCCTTTTTT |

**Table S2. Primer sequences**

| Primer | Forward (5’ to 3’) | Reverse (5’ to 3’) |
| --- | --- | --- |
| Gapdh | AGGTCGGTGTGAACGGATTTG | TGTAGACCATGTAGTTGAGGTCA |
| Pparg | GGAAGACCACTCGCATTCCTT | TCGCACTTTGGTATTCTTGGAG |
| Cidea | ATCACAACTGGCCTGGTTACG | TACTACCCGGTGTCCATTTCT |
| Cidec | CCTATGACCTGCACTGCTACAAG | CATGTAGCTGGAGGTGCCAAG |
| Plin2 | GCGGACTTGCTCGTCCCTCA | GGTCACGGTCTTCACGCCCT |
| Hilpda | TGCTGGGCATCATGTTGACC | TGACCCCTCGTGATCCAGG |
| Cd36 | AATGGCACAGACGCAGCCT | GGTTGTCTGGATTCTGGA |

**Table S3. PPAR Target genes**

|  | PPARα | PPARβ/δ | PPARγ | References |
| --- | --- | --- | --- | --- |
| **Fatty acid oxidation** |  |  |  |  |
| ACAA1 | * | * | * | (1); (2); (1) |
| ACAA2 | * | * | * | (1); (2); (1) |
| ACADM | * |  |  | (3) |
| ACADVL | * | * | * | (3); (2); (1) |
| ACOT1 |  | * |  | (3) |
| ACOT8 | * |  | * | (1); (1) |
| ACOX1 | * | * | * | (3); (2); (1) |
| ACS | * | * | * | (1); KEGG; KEGG |
| ACSL1 | * | * |  | (3); (2) |
| ACSL3 |  | * |  | (4) |
| CPT | * |  |  | (2) |
| CPT1A | * | * | * | (3); (3); (1) |
| CPT1B | * | * | * | (1); (2); (1) |
| CPT2 | * | * | * | (5); (2); (1) |
| FABP1 | * | * | * | (3); (2); (1) |
| FABP2 | * |  | * | (1); (1) |
| FABP3 | * |  |  | (1) |
| FABP5 | * | * | * | (1); (2); (1) |
| MCAD |  | * |  | (3) |
| MLYCD | * | * | * | (1); (2); (1) |
| PGC-1α |  | * |  | (3) |
| SCPX | * | * | * | KEGG |
| SLC25A20 | * | * | * | (3); (2) |
|  |  |  |  |  |
| **Fatty acid synthesis** |  |  |  |  |
| ACACA |  | * | * | (3); (3) |
| ACACB |  | * |  | (3) |
| ACLY |  | * | * | (3); (3) |
| ELOVL4 |  |  | * | (6) |
| FADS2 | * |  | * | (5); KEGG |
| FASN |  | * | * | (3); (3) |
| SCD1 | * |  | * | KEGG; (3) |
| SCD2 |  |  | * | (7) |
|  |  |  |  |  |
| **Fatty acid transport** |  |  |  |  |
| OLR1 | * |  |  | KEGG |
|  |  |  |  |  |
| **Lipolysis** |  |  |  |  |
| LIPE |  | * | * | (2); (1) |
| LPIG | * | * |  | (1); (2) |
|  |  |  |  |  |
| **Triglyceride synthesis** |  |  |  |  |
| CD36 | * |  | * | (2); (3) |
| CIDEC |  |  | * | (8) |
| FABP4 |  |  | * | (3) |
| FSP27 |  |  | * | (3) |
| GK |  |  | * | KEGG |
| GPAM |  | * | * | (2); (1) |
| GPD1 |  | * | * | (2); (1) |
| HILPDA |  |  | * | (9) |
| INSIG1 |  | * | * | (10); (11) |
| LPIN1 |  |  | * | (12) |
| MOGAT1 |  |  | * | (3) |
| PLIN2 |  |  | * | (3) |
| SLC27A1 | * | * |  | (2); (2) |
| SREBF1 | * | * |  | (1); (2) |
| SLC27A4 | * |  | * | (1); (1) |
|  |  |  |  |  |
| **Ketogenesis** |  |  |  |  |
| HMGCS2 | * |  |  | (2) |
|  |  |  |  |  |
| **Glycogenolysis** |  |  |  |  |
| AQP7 |  |  | * | KEGG |
| ME1 | * | * | * | KEGG; (3); KEGG |
| PGK1 |  |  | * | (13) |
| PEPCK |  | * | * | (3); (14) |
| PGD |  | * |  | (3) |
|  |  |  |  |  |
| **Glycolysis** |  |  |  |  |
| GAPDH | * |  |  | (2) |
| GCK |  | * | * | (2); (15) |
| HK1 |  |  | * | (2) |
| PFKP |  |  | * | (16) |
| PK |  | * | * | (2); (2) |
| SLC2A2 |  | * |  | (2) |
|  |  |  |  |  |
| **Cyp450s** |  |  |  |  |
| CYP7A1 | * |  | * | KEGG; KEGG |
| CYP8B1 | * |  | * | KEGG; KEGG |
| CYP27 | * |  | * | KEGG; KEGG |
| CYP24A1 | * | * | * | KEGG; KEGG; KEGG |
|  |  |  |  |  |
| **Lipoprotein synthesis** |  |  |  |  |
| APOC3 | * |  |  | (2) |
| APOA4 | * | * |  | (2); (2) |
| APOC3 |  | * |  | (2) |
| APOE | * |  |  | (1) |
| LDLR |  | * |  | (17) |
| VLDLR |  | * | * | (3); (18) |

* Indicates the existence of relevant literature reports pertaining to PPAR target genes.

**Table S4. Molecular docking results**

| Name | Structure | PPARα-6KBA  (kcal·mol^-1^) | PPARβ/δ-3TKM  (kcal·mol^-1^) | PPARγ-5YCP  (kcal·mol^-1^) |
| --- | --- | --- | --- | --- |
| Pirinixic acid  (Wy-14643) |  | -9.98 |  |  |
| GW0742 |  |  | -12.39 |  |
| Rosiglitazone |  |  |  | -9.64 |
| Benzbromarone |  | -6.85 | -7.29 | -7.58 |
| 6-Hydroxybenzbromarone |  | -7.69 | -7.13 | -7.76 |
| 5,6-dihydroxybenzbromarone |  | -7.24 | -6.53 | -8.23 |
| 6,7-dihydroxybenzbromarone |  | -6.90 | -6.99 | -6.26 |
| URC-102 |  | -7.6 | -7.3 | -6.8 |
| Dotinurad  (FYU-981) |  | -8.6 | -7.7 | -7.7 |
| Xininurad  (XNW-3009) |  | -7.8 | -8.0 | -8.4 |
| AR882 |  | -9.5 | -9.6 | -9.6 |
| ABP671 |  | -8.2 | -7.8 | -8.5 |

**References**

1. Cheng HS, Tan WR, Low ZS, Marvalim C, Lee JYH, Tan NS. Exploration and Development of PPAR Modulators in Health and Disease: An Update of Clinical Evidence. Int J Mol Sci 2019;20.

2. Lange NF, Graf V, Caussy C, Dufour JF. PPAR-Targeted Therapies in the Treatment of Non-Alcoholic Fatty Liver Disease in Diabetic Patients. Int J Mol Sci 2022;23.

3. Wang Y, Nakajima T, Gonzalez FJ, Tanaka N. PPARs as Metabolic Regulators in the Liver: Lessons from Liver-Specific PPAR-Null Mice. Int J Mol Sci 2020;21.

4. Cao A, Li H, Zhou Y, Wu M, Liu J. Long chain acyl-CoA synthetase-3 is a molecular target for peroxisome proliferator-activated receptor delta in HepG2 hepatoma cells. J Biol Chem 2010;285:16664-16674.

5. Contreras AV, Torres N, Tovar AR. PPAR-α as a key nutritional and environmental sensor for metabolic adaptation. Adv Nutr 2013;4:439-452.

6. Igarashi T, Yanagi H, Yagi M, Ichihashi M, Imokawa G. Horse-Derived Ceramide Accentuates Glucosylceramide Synthase and Ceramide Synthase 3 by Activating PPARβ/δ and/or PPARγ to Stimulate Ceramide Synthesis. Biomedicines 2023;11.

7. Kim YC, Gomez FE, Fox BG, Ntambi JM. Differential regulation of the stearoyl-CoA desaturase genes by thiazolidinediones in 3T3-L1 adipocytes. J Lipid Res 2000;41:1310-1316.

8. Kim YJ, Cho SY, Yun CH, Moon YS, Lee TR, Kim SH. Transcriptional activation of Cidec by PPARgamma2 in adipocyte. Biochem Biophys Res Commun 2008;377:297-302.

9. de la Rosa Rodriguez MA, Kersten S. Regulation of lipid droplet-associated proteins by peroxisome proliferator-activated receptors. Biochim Biophys Acta Mol Cell Biol Lipids 2017;1862:1212-1220.

10. Qin X, Xie X, Fan Y, Tian J, Guan Y, Wang X, Zhu Y, et al. Peroxisome proliferator-activated receptor-delta induces insulin-induced gene-1 and suppresses hepatic lipogenesis in obese diabetic mice. Hepatology 2008;48:432-441.

11. Kast-Woelbern HR, Dana SL, Cesario RM, Sun L, de Grandpre LY, Brooks ME, Osburn DL, et al. Rosiglitazone induction of Insig-1 in white adipose tissue reveals a novel interplay of peroxisome proliferator-activated receptor gamma and sterol regulatory element-binding protein in the regulation of adipogenesis. J Biol Chem 2004;279:23908-23915.

12. Finck BN, Gropler MC, Chen Z, Leone TC, Croce MA, Harris TE, Lawrence JC, Jr., et al. Lipin 1 is an inducible amplifier of the hepatic PGC-1alpha/PPARalpha regulatory pathway. Cell Metab 2006;4:199-210.

13. Shashni B, Sakharkar KR, Nagasaki Y, Sakharkar MK. Glycolytic enzymes PGK1 and PKM2 as novel transcriptional targets of PPARγ in breast cancer pathophysiology. J Drug Target 2013;21:161-174.

14. Chen H, Tan H, Wan J, Zeng Y, Wang J, Wang H, Lu X. PPAR-γ signaling in nonalcoholic fatty liver disease: Pathogenesis and therapeutic targets. Pharmacol Ther 2023;245:108391.

15. Kim HI, Ahn YH. Role of peroxisome proliferator-activated receptor-gamma in the glucose-sensing apparatus of liver and beta-cells. Diabetes 2004;53 Suppl 1:S60-65.

16. Calvier L, Chouvarine P, Legchenko E, Hoffmann N, Geldner J, Borchert P, Jonigk D, et al. PPARγ Links BMP2 and TGFβ1 Pathways in Vascular Smooth Muscle Cells, Regulating Cell Proliferation and Glucose Metabolism. Cell Metab 2017;25:1118-1134.e1117.

17. Shende VR, Singh AB, Liu J. A novel peroxisome proliferator response element modulates hepatic low-density lipoprotein receptor gene transcription in response to PPARδ activation. Biochem J 2015;472:275-286.

18. Hajri T, Zaiou M, Fungwe TV, Ouguerram K, Besong S. Epigenetic Regulation of Peroxisome Proliferator-Activated Receptor Gamma Mediates High-Fat Diet-Induced Non-Alcoholic Fatty Liver Disease. Cells 2021;10.
